# Prevalence and factors associated with hepatitis C among pregnant women in China: a cross-sectional study

**DOI:** 10.1038/s41598-023-27668-3

**Published:** 2023-01-14

**Authors:** Sun He, Gao Shuang, Wu Yinglan, Wang Lan, Wang Wei, Wang Ailing, Wang Changhe, Wang Xiaoyan, Gao Qun, Lu Zechun, Huang Dongxu, Wang Yu, Mo Phoenix Kit Han, Chen Zhongdan, Polin Chan, Wang Qian

**Affiliations:** 1 National Center for Women and Children’s Health, Chinese Center for Disease Control and Prevention, Beijing, China; 2 Guangdong Women and Children Hospital, Guangdong, China; 3 Hunan Provincial Maternal and Child Health Care Hospital, Changsha, China; 4 Chongqing Health Center for Women and Children, Chongqing, China; 5 Shenzhen Baoan Women’s and Children’s Hospital, Guangdong, China; 6 School of Public Health and Primary Care, The Chinese University of Hong Kong, Sha Tin, Hong Kong China; 7 WHO China Office, Beijing, China; 8 WHO West Pacific Regional Office, Manila, Philippines

**Keywords:** Diseases, Risk factors

## Abstract

Pregnant women infected with HCV should be given attention due to their special physiological stage and the effect on offspring health. To examine the prevalence of HCV infection among pregnant women in part of China and explore relevant factors during pregnancy, a cross-sectional study was conducted in four maternal and children health care institutions (MCHC) in Guangdong, Hunan and Chongqing. Pregnant women who were delivered, induced or spontaneous abortion were included and relevant information was collected through the Hospital Information System. Results showed that the prevalence of HCV among pregnant women in four MCHCs was 0.11% (95% CI 0.09–0.13%). Age, occupations, regions, syphilis-infection, intrahepatic cholestasis of pregnancy (ICP), and placenta previa were significant factors (all *P* < 0.05). Age and syphilis-infection were positively correlated with HCV infection (*Z* = 3.41, *P* = 0.0006; OR = 18.16, 95% CI 9.34–35.29). HCV and HBV infection were risk factors of ICP (OR = 4.18, 95% CI 2.18–8.04; OR = 2.59, 95% CI 2.31–2.89). Our study indicates that the prevalence of HCV among pregnant women in the three provinces(city) was low compared with the general population in China. Older age and syphilis-infection increased the risk of HCV infection during pregnancy. HCV infection was a risk factor of ICP. Generally, we need keep a watchful eye on HCV infection and relevant factors mentioned above during pregnancy in clinic, especially those also infected with syphilis. HCV testing based on risk factors is recommended in antenatal care and obstetrics.

## Introduction

Hepatitis C, caused by hepatitis C virus (HCV) is an infectious disease with clear transmission routes and insidious onset. According to the World Health Organization (WHO), about 58 million people were infected with HCV globally in 2019, accounting for 0.75% of the total population^1^. Worldwide, up to 8% of pregnant women are infected by HCV^2^. Mother-to-child transmission is one of the main transmission routes of HCV^3^, with the rate of 5.8% (95% CI 4.2%–7.8%) worldwide^4^. As a curable chronic infectious disease, perinatal hepatitis C infection has a spontaneous clearance rate (25–30%)^5^, so mother-to-child transmission of HCV has been ignored. However, in fact, once diagnosed with HCV infection, more than 80% of newborns will show chronic symptoms^6^, in which 30% will develop clinical symptoms in childhood or adulthood, and are at high risk of liver cirrhosis and hepatocellular carcinoma (HCC)^7^. In addition, hepatitis C infection is strongly associated with adverse pregnancy outcomes, including fetal growth restriction, low birth weight, congenital abnormalities, and preterm birth^4^. Therefore, understanding the prevalence of HCV among pregnant women and exploring risk factors should be given high importance.

In 2016, WHO proposed the goal of eliminating viral hepatitis as a public health threat by 2030^8^—a 90% reduction in new infections and a 65% reduction in mortality. Whereas most studies on viral hepatitis infection primarily focused on HBV^9^ or specially HCV exposed populations such as people who inject drugs (PWID) and men who have sex with men (MSM)^10–12^, studies among pregnant women were scarce. To achieve the goal of eliminating HCV as a public health threat, reducing the disease burden of society and decreasing new HCV infections, study on HCV pregnant women and HCV mother-to-children transmission is essential. In this study, we investigated the pregnant women who had been admitted in selected maternal and children health care institutions (MCHC) in some regions of China by collecting data from the Hospital Information System (HIS), so as to understand the prevalence of HCV infection among pregnant women and its associated factors.

## Participants and method

### Study sites and participants

**Figure 1:**
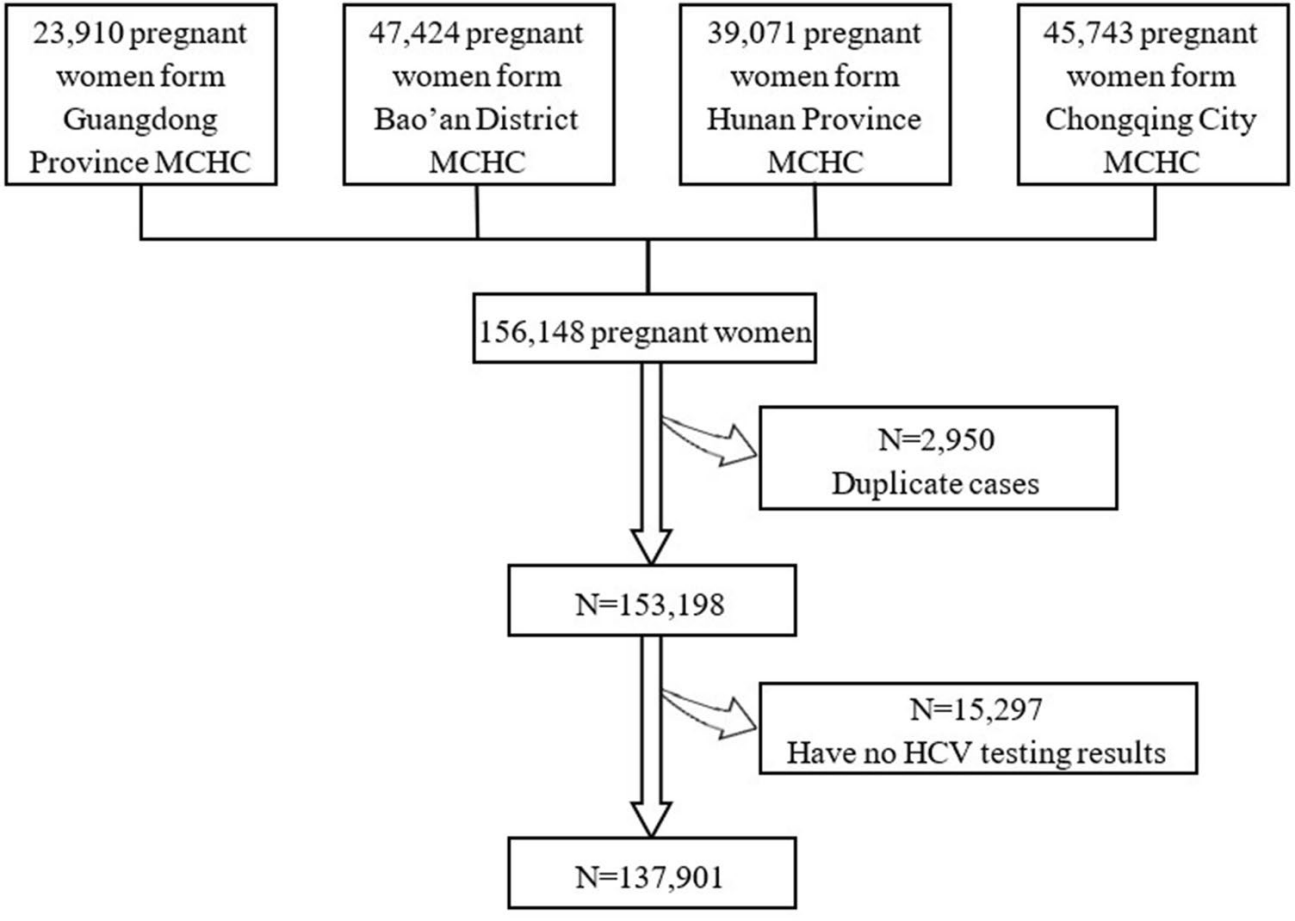
Flowchart of study participants.

Taking into account the high number of annual births, good socio-economic level, similar health conditions, and wide coverage of services, we performed a cross-sectional study in four sites, i.e. Guangdong Province MCHC, Bao’an District MCHC, Chongqing City MCHC, and Hunan Province MCHC. We included 156,148 pregnant women who were admitted to the selected hospital for delivery from 1 July 2019 to 31 December 2021 with birth outcomes including natural vaginal birth, cesarean section and induction, of whom 2950 duplicate cases and 15,297 cases without HCV testing results were excluded. Overall, 137,901 cases were contained into the final dataset. Details about the flow of participants recruitment were listed in Fig. 1. This study has been reviewed by the Ethics Committee of National Center for Women and Children’s Health, Chinese Center for Disease Control and Prevention (Approval No.: FY2021-16). Informed consent was obtained from all the participants.

### Method

All the data were collected from the Hospital Information System (HIS) of obstetrics, hospital laboratory or infection department in the study sites. The investigators were trained uniformly and arranged to export information in the same way. Participants were not involved in the design, or conduct, or reporting, or dissemination plans of our research. All methods were performed in accordance with the relevant guidelines and regulations. Designed by National Center for Women and Children's Health, Chinese Center for Disease Control and Prevention, and completed by trained collectors from the study sites, the registration form contained information about maternal sociodemographic characteristics, delivery results, the newborn status, maternal HCV test time and results. Information about the newborn included newborn defects, gender and weight. Maternal HCV test time was defined as the gestational week of the last test before delivery. Laboratory tests were carried out by the ELISA (enzyme-linked immunoassay) method to identify the presence of anti-HCV antibodies, the specific operation was carried out strictly according to the kit steps from Beijing Wantai Biological Pharmaceutical Company.

### Statistical analyses

Quantitative variables were expressed as mean ± SD, and analyzed using student’s t-test. Categorical variables were expressed as frequencies and percentages. The *χ*^2^ test and exact Fishers test were applied for the analysis of categorical variables. Statistical significance level was set at a two-sided p < 0.05. Stepwise logistic regression was performed to identify associated factors of HCV infection. To explore whether HCV infection may exacerbate the occurrence of adverse pregnancy outcomes or related complications, model fitness was tested using multivariate logistic regression. All the analyses were performed on SAS 9.4.

### Ethical approval

This study has been approved by the Ethics Committee of Maternal and Child Health Center, Chinese Center for Disease Control and Prevention (Approval No.: FY2021-16).

## Results

### Sociodemographic characteristics

As HCV screening has not been included into the scope of routine prenatal health care in China, the linked database contained 15,297 cases without HCV testing results and the prenatal screening rate was 90.20% among the four study sites. After excluding those duplicate cases and those without HCV testing results, the total sample consisted of 137,901 individuals, with ages ranging from 13 to 59 (30.30 ± 4.14). Of these, 20,492 were elder than 35 years old (14.86%), and 273 were younger than 20 years old (0.20%). Most individuals were unemployed (33.14%) and national staff (39.25%). As for the regional distribution, participants were from three provincial regions, namely Guangdong (38.50%), Hunan (28.33%) and Chongqing (33.17%). The majority of them were the Han nationality (96.03%), see in Table 1.

**Table 1:** HCV antibody positive rate of pregnant women.

Classification	Total	HCV positive		*χ*^*2*^/*P*
		No	Rates (%)	
**Participants**	137,901	152	0.11 (0.09–0.13)	–
**Age groups**				25.69/0.0001
< 20	273	0	0.00	
20–24	9270	3	0.03	
25–29	52,662	60	0.11	
30–34	55,204	47	0.09	
35–39	17,563	35	0.20	
≥ 40	2929	7	0.24	
**Occupation**				21.70/0.0099
Workers	1274	1	0.08	
National staff	54,120	45	0.08	
Students	191	0	0.00	
Professionals	14,148	7	0.05	
Service workers	161	1	0.62	
Enterprise managers	2605	2	0.08	
Individual operators	4901	7	0.14	
Freelancers	5421	9	0.12	
Others	9362	11	0.17	
Unemployed	45,698	69	0.15	
**Region**				22.57/ < 0.0001
Guangdong Province	53,087	87	0.16	
Hunan Province	39,071	30	0.08	
Chongqing City	45,743	35	0.08	
**Ethnic**				0.11/0.95
Han	123,665	133	0.11	
Minorities	5057	6	0.12	
Foreigners	53	0	0.00	

### Prevalence of HCV among pregnant women in different dimension

**Figure 2:**
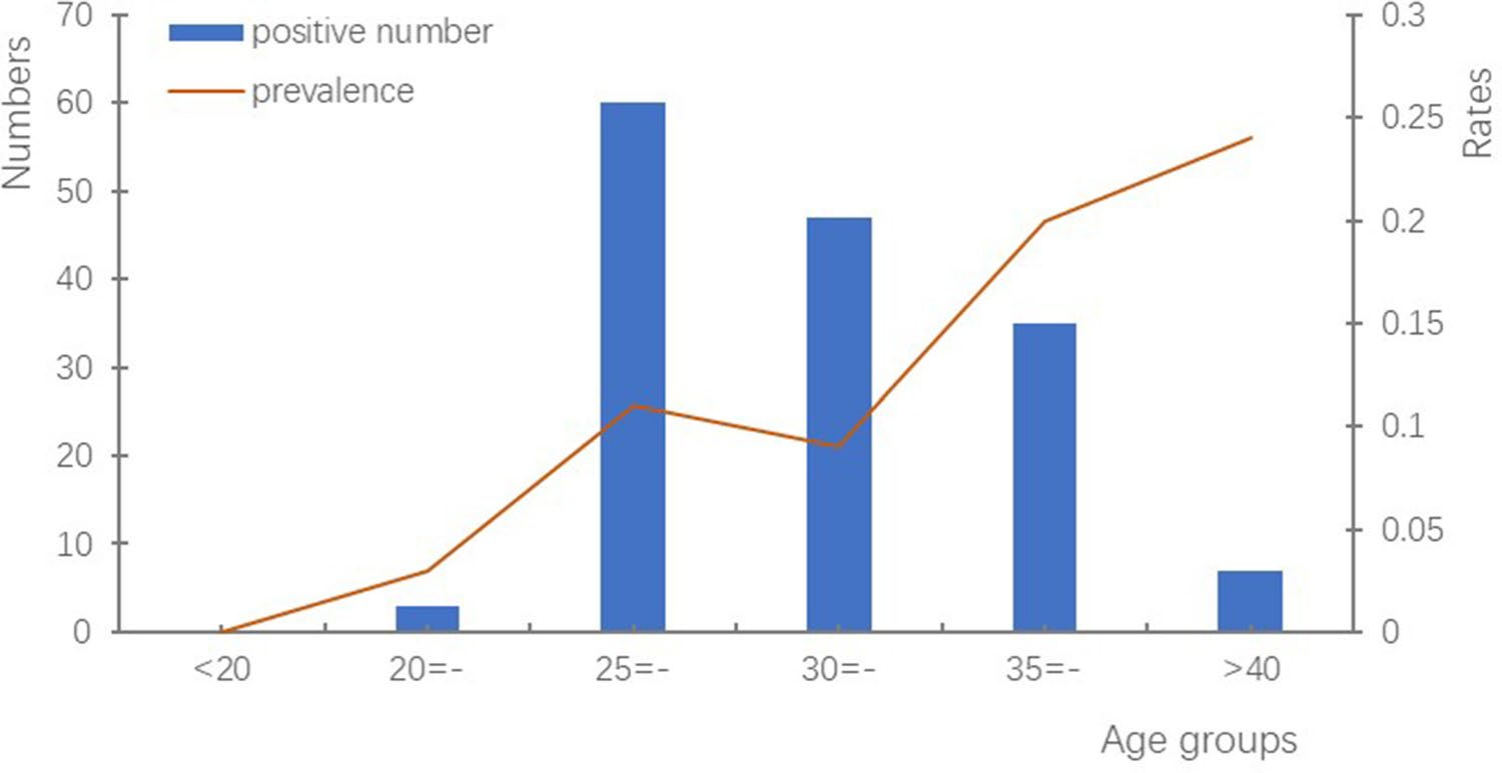
Trend of HCV prevalence with age in four MCHC.

The positive rate of HCV antibody among the participants was 0.11% (95% CI 0.09%–0.13%). As shown in Table 1 and Fig. 2, the prevalence appeared to be higher among older individuals (Cochran–Armitage test *Z* = 3.41, *P* = 0.0006) and the highest was found among participants who were aged above 40 years (0.24%), with statistical significance (*χ*^*2*^ = 25.69, *P* = 0.0001). In addition, service workers had a higher HCV prevalence (0.62%) than other occupations, with statistical significance (*χ*^*2*^ = 21.70, *P* = 0.0099). Meanwhile, Guangdong Province has the highest prevalence (*χ*^*2*^ = 22.57, *P* < 0.0001) across the study regions.

### HCV and syphilis or HBV co-infection

A number of HCV-infected patients with syphilis or HBV co-infection was identified. There were 5 pregnant women co-infected HCV and HBV, 9 co-infected HCV and syphilis and 2 infected all three viruses simultaneously. Therefore, the co-infection rate of HBV and syphilis among HCV-infected patients was 4.86% and 7.64% respectively.

### Factors of HCV infection in pregnant women

Results from *χ*^*2*^ test and Fisher test reported that syphilis infection (*χ*^*2*^ = 164.51, *P* < 0.0001), intrahepatic cholestasis of pregnancy (ICP) (Fishers test *P* = 0.0009), and placenta previa (Fishers test *P* = 0.03) were significantly associated with HCV infection (Table 2). Other factors, including gestational hypertension, diabetes, eclampsia, fetal distress, premature rupture of membranes (PROM) and premature delivery did not reach statistical significance. Results from the stepwise logistic regression model showed that those over the age of 25 were at a higher risk of contracting HCV (OR = 3.04, 95 CI 0.95–9.75 for 25–29; 2.23, 0.69–7.22 for 30–34; 5.32, 1.63–17.39 for 35–39; 7.02, 1.81–27.23 for 40 or above). Furthermore, compared with participants from Guangdong Province, those from Hunan province (OR = 0.49, 95% CI 0.31–0.75) and Chongqing city (OR = 0.48, 95% CI 0.32–0.73) have a significantly lower risk of HCV infection. Syphilis infection was associated with higher risk of HCV infection (OR = 18.16, 95% CI 9.34–35.29) (Table 3). Results from the multivariate logistic regressions indicated that HCV and HBV infection could increase the risk of ICP (OR = 4.18, 95% CI 2.18–8.04; OR = 2.59, 95% CI 2.31–2.89) while HCV infection seemed to have no influence on other complications and outcomes (Table 4).

**Table 2:** Univariate analyses of HCV associated factors among pregnant women in three provinces.

Classification	HCV positive (+)		HCV negative (−)		Statistical value	*P* value
	No	Percentage(%)	No	Percentage(%)		
**HBV**					0.65^a^	0.42
Positive ( +)	7	4.86	8902	6.52		
Negative (−)	137	95.14	127,631	93.48		
**Syphilis**					–	< 0.0001^b^
Positive ( +)	11	7.64	610	0.45		
Negative (−)	133	92.36	135,923	99.55		
**ICP**					–	0.0009^b^
Yes	10	6.94	2844	2.08		
No	134	93.76	133,689	97.92		
**Gestational hypertension**					0.76^a^	0.38
Yes	8	5.56	5606	4.11		
No	136	94.44	130,927	95.89		
**Gestational diabetes**					0.91^a^	0.34
Yes	35	24.31	28,736	21.05		
No	109	75.69	107,797	78.95		
**Gestational anemia**					2.93^a^	0.08
Yes	40	27.78	47,196	34.57		
No	104	72.22	89,337	65.43		
**Eclampsia**					0.40^a^	0.53
Yes	6	4.17	4412	3.23		
No	138	95.83	132,121	96.77		
**Placenta previa**					–	0.03^b^
Yes	6	4.17	2209	1.68		
No	138	95.83	134,243	98.32		
**Fetal distress**					0.02^a^	0.90
Yes	16	11.11	15,631	11.45		
No	128	88.89	120,902	88.55		
**Premature delivery**					0.50^a^	0.47
Yes	12	8.33	9336	6.84		
No	132	91.67	127,197	93.16		
**Premature rupture of membranes (PROM)**					0.0035^a^	0.95
Yes	37	25.69	34,790	25.48		
No	107	74.31	101,743	74.52		
**Birth weight **($\overline{x }\pm s$)	3147.91 ± 549.07		3217.56 ± 489.55		− 1.68^c^	0.09

^a^*χ*^*2*^ value.

^b^Fisher test.

^c^t-test value.

**Table 3:** Multivariate Logistic regression model analysis of HCV associated factors among pregnant women in three provinces.

Classification	*β* value	Standard error	Wald *χ*^*2*^	*P* value	*OR* value (95% CI )
**Regions**					
Guangdong Province					1.00
Hunan Province	− 0.73	0.22	10.70	0.0011	0.49 (0.31–0.75)
Chongqing City	− 0.73	0.21	11.91	0.0006	0.48 (0.32–0.73)
**Age**					
< 25					1.00
25–29	1.11	0.59	3.49	0.0617	3.04 (0.95–9.75)
30–34	0.80	0.60	1.79	0.1810	2.23 (0.69–7.22)
35–39	1.67	0.61	7.63	0.0057	5.32 (1.63–17.39)
≥ 40	1.95	0.69	7.93	0.0049	7.02 (1.81–27.23)
**Syphilis**^a^	2.90	0.34	73.11	< 0.001	18.16 (9.34–35.29)

^a^"No" was used as the reference group.

**Table 4:** Multivariate logistic regression model analysis of associated factors of ICP among pregnant women in three provinces.

Classification	*β* value	Standard error	Wald *χ*^*2*^	*P* value	*OR* value (95% CI )
**Regions**					
Guangdong Province					1.00
Hunan Province	− 0.48	0.07	41.54	< 0.0001	0.62 (0.54–0.72)
Chongqing City	0.76	0.05	208.31	< 0.0001	2.15 (1.93–2.38)
**Work**					
Workers	− 1.04	0.34	9.52	0.0020	0.34 (0.18–0.68)
National staff	− 0.14	0.05	7.23	0.0072	0.87 (0.78–0.96)
Students	0.32	0.46	0.48	0.4896	1.37 (0.56–3.38)
Professionals	− 0.03	0.07	0.16	0.6882	0.97 (0.84–1.12)
Service workers	− 10.73	151.7	0.01	0.9436	0.01 (0.01–99.99)
Enterprise managers	0.06	0.17	0.15	0.7033	1.07 (0.77–1.49)
Individual operators	− 0.17	0.12	1.90	0.1680	0.85 (0.67–1.07)
Freelancers	− 0.003	0.11	0.0009	0.9755	0.99 (0.81–1.23)
Others	− 0.13	0.10	1.82	0.1777	0.88 (0.73–1.06)
Unemployed					1.00
HCV^a^	1.19	0.42	7.82	0.0052	3.28 (1.43–7.55)
HBV^a^	1.03	0.06	266.19	< 0.0001	2.79 (2.47–3.16)
Gestational hypertension^a^	0.42	0.09	22.31	< 0.0001	1.52 (1.28–1.81)
Gestational anemia^a^	0.11	0.05	5.78	0.0162	1.12 (1.02–1.22)

^a^"No" was used as the reference group.

## Discussion

Hepatitis C virus infection may lead to cirrhosis, liver failure, and hepatocellular carcinoma, it will also transmit from mother to offspring^13^. Thus, it is vitally important to investigate the prevalence of HCV infection among pregnant women. Nowadays, routine HCV screening is currently not widely recommended during pregnancy for the reason of cost-effectiveness and the evidence of spontaneous clearance in Japan, Canada, China and some other countries^14–16^. From the results we analyzed, the HCV prenatal screening rate during pregnancy was 90.20%, which was lower than the rate of hepatitis B virus, HIV and syphilis during pregnancy in China^17–19^.

In this study, the positive rate of HCV antibody in pregnant women is 0.11% (95% CI 0.09%–0.13%), which is similar to those reported in local studies. For example, surveillance data from Zhongshan city in Guangdong province showed that the proportion of pregnant women infected with HCV gradually decreased from 2009 to 2019, and the HCV positive rate in 2018 was 0.25%, 0.00% in 2019^20^. A meta-analysis by Ma et al. on the positive rate of hepatitis C antibody among pregnant women in China from 2008 to 2018 showed the rate was 0.235% (95% CI 0.189%–0.286%)^21^. In the general population in China, the prevalence of HCV is 0.38% (95% CI 0.23%–0.53%)^22^, which is higher than what we found in pregnant women. While compared with other counties, the rate seems to be close to developed countries but lower than low and middle-income ones. The reported prevalence of maternal hepatitis C virus infection in the United States was 0.24% in 2020 while such rate was much higher in Africa^23^ (3.4%), Pakistan^24^ (2.22%) and Egypt^25^ (6.1%).

It would be noteworthy to identity those sociodemographic characteristics that were associated with higher risk of HCV infection. Older age was found to be a significant factor of HCV positive rate in the present study (Cochran–Armitage test *Z* = 3.41, *P* = 0.0006). Similarly, Khamis et al.^25^ and Costa et al.^26^ found that older age was one of the most important risk factors for HCV infection among pregnant women. However, Dagnew et al.^27^ reported a higher HCV prevalence among young pregnant women, attributing to the fact that young females are more active sexually and more probable to expose to multiple sex. Our findings also showed that region and occupation were significant factors of HCV infection. This could be attributed to differences in the geographical location, various socioeconomic status and lack of awareness of HCV infection among certain regions.

Analyses of gestational complications and adverse neonatal outcomes in relation to HCV infection during pregnancy can be complex as it’s hard to discern whether the infection is a direct influence or a potential confounding factor. Nevertheless, we confirmed that HCV infection was found to be one of the risk factors of ICP during pregnancy, which was in agreement with the extant literature that indicated^28–30^ that ICP was more prevalent in patients, including pregnant women, with chronic HCV infection. Besides, no association between gestational diabetes and HCV infection was observed in our study, whereas Samir Rouabhia et al.^31^ believed that HCV exacerbated insulin resistance or diabetes leading to a reduced ability to fight infection, leading to an increased risk of type 2 diabetes among those with HCV infection during pregnancy. We also found placenta previa and HCV infection were related but HCV infection had no effect on the occurrence of placenta previa. Similarly, Piffer et al.^32^ found no significance for eclampsia, premature rupture of membranes and placenta previa. It is reported^33,34^ that the adverse neonatal outcomes like preterm birth and low birth weight (LBW) were more likely to occur in mothers with HCV, however such finding was not replicated in the present study.

It is intriguing that despite the evidence that coinfection with sexually transmitted diseases^34–37^ could aggregate the prevalence of HCV due to the similar transmission way and similar pathological mechanism, such association was not significant in the present study in terms of HBV co-infection and there were no cases with HCV and HIV co-infection. While syphilis co-infection was identified as a risk factor from the data analysis, the co-infection rate of HBV and syphilis in HCV-infected participants was 4.86% and 7.64%, respectively. The coinfection rate was diverse from those reported in the extant literature^38,39^, which reports a HBV rate of 5.7% in hemodialyzers with HCV infection, a HIV rate of 3.9% in pregnant women, and syphilis rate of 0.5% in unpaid blood donors. The possible reasons for such discrepancies may be that the participants were mostly from the urban areas, with possibility higher level of education, health awareness and financial situation. From the perspective of the clinic, pregnant women who were coinfected with sexually transmitted diseases should be managed and taken seriously. The combination of screening and follow-up monitoring should be taken into consideration.

There were some limitations that should be noted. Firstly, data were only collected from three provinces or cities due to time and funding limitations, therefore findings may not be generalized to the whole pregnant women population in China. In addition, the present study was cross-sectional in nature, which cannot reflect the casual relationship. Meanwhile, there were duplicates (2950) and cases without HCV testing results (15,297) during data collection, which may be due to insufficient on-site supervision and the low screening rate of prenatal HCV. Indeed, HCV prenatal screening could help to know the prevalence of HCV among pregnant women and their hepatitis C virus status, which facilitates the clinical identification and management of key maternal outcomes timely. More studies on cost-effectiveness and disease burden is needed to decide whether universal screening of HCV is recommended in the clinic.

Findings from the present and previous studies^14^ indicate that HCV testing based on the presence of risk factors may be warranted among pregnant women in antenatal care clinic as well as obstetrics department. Besides, prevention and health care knowledge about HCV should be designed to educate and promote in communities, hospitals, schools, etc. Moreover, longitudinal studies on HCV infection among pregnant women should be carried out based on data support of this study to understand the mechanism of mother-to-child transmission of HCV, explore the prevention of neonatal infection with HCV, and clarify the risk factors of HCV infection. In order to reach the WHO goal of HCV elimination, efforts and support from all sectors of society are necessary.

## Conclusion

The prevalence of HCV infection among pregnant women in Guangdong province, Hunan province and Chongqing city was 0.11% (95% CI 0.09%–0.13%). This indicator was at a low level compared with the general population in China. Age, region and syphilis infection were significantly associated with HCV infection during pregnancy. And HCV infection was found to be one of risk factors of intrahepatic cholestasis of pregnancy (ICP). In conclusion, pregnant women with HCV should be paid high attention in clinic, especially those infected with syphilis at the same time. HCV testing based on risk factors is recommended so as to protect the health of pregnant women and their infants.

## Data Availability

The data presented in this study are available on request from the corresponding author.

## Abbreviations

HCV: Hepatitis C virus
MCHC: Maternal and children health care institution
ICP: Intrahepatic cholestasis of pregnancy
WHO: World Health Organization
PWID: People who inject drugs
MSM: Men who have sex with men
HIS: Hospital Information System
ELISA: Enzyme-linked immunoassay
PROM: Premature rupture of membranes
LBW: Low birth weight

## References

[1] World Health, O. *Global Progress Report on HIV, Viral Hepatitis and Sexually Transmitted Infections, 2021: Accountability for the Global Health Sector Strategies 2016–2021: Actions for Impact*. xii, 92 p. (World Health Organization, 2021).

[2] Spera AM, Eldin TK, Tosone G, Orlando R (2016). Antiviral therapy for hepatitis C: Has anything changed for pregnant/lactating women?. World J. Hepatol..

[3] Wang W (2021). Research progress on the latest transmission route and prevention of hepatitis C. Cap. Med..

[4] Benova L, Mohamoud YA, Calvert C, Abu-Raddad LJ (2014). Vertical transmission of hepatitis C virus: Systematic review and meta-analysis. Clin. Infect. Dis..

[5] Wen ZX, Zhu P, Wang YM (2012). Recent progress in clinical research of hepatitis C virus infection in pediatric patients. J. Clin. Hepatol..

[6] Guido M, Bortolotti F (2011). Viral hepatitis: Treating hepatitis C in children: An open horizon. Nat. Rev. Gastroenterol. Hepatol..

[7] González-Peralta RP (2009). Hepatocellular carcinoma in 2 young adolescents with chronic hepatitis C. J. Pediatr. Gastroenterol. Nutr..

[8] World Health, O. *Global Health Sector Strategy on Viral Hepatitis 2016–2021. Towards Ending Viral Hepatitis* (World Health Organization, Geneva, 2016).

[9] Davlidova S (2021). Prevalence of HIV, HCV and HBV in Central Asia and the Caucasus: A systematic review. Int. J. Infect. Dis..

[10] Fu Y (2011). New trends of HCV infection in China revealed by genetic analysis of viral sequences determined from first-time volunteer blood donors. J. Viral Hepat..

[11] Lee CY, Wu PH, Lu MW, Chen TC, Lu PL (2021). High prevalence of unawareness of HCV infection status among both HCV-seronegative and seropositive people living with human immunodeficiency virus in Taiwan. PLoS One.

[12] Kim KA (2021). Epidemiology and treatment status of hepatitis C virus infection among people who have ever injected drugs in Korea: A prospective multicenter cohort study from 2007 to 2019 in comparison with non-PWID. Epidemiol. Health.

[13] Kushner T, Reau N (2021). Changing epidemiology, implications, and recommendations for hepatitis C in women of childbearing age and during pregnancy. J. Hepatol..

[14] McDermott CD, Moravac CC, Yudin MH (2010). The effectiveness of screening for hepatitis C in pregnancy. J. Obstet. Gynaecol. Can..

[15] Nagai K (2020). Estimating the cost-effectiveness of screening for hepatitis C virus infection in Japan. Hepatol. Res..

[16] Wang Y, Mao YR (2021). Current status of nucleic acid testing for hepatitis C. Chin. J. Hepatol..

[17] Yu XX (2020). Analysis on the detection of AIDS, syphilis and HBV among pregnant women in Changzhou city in 2018. Chin. J. Woman Child Health Res..

[18] National Health Commission. *Letter of Reply to Proposal No. 0703 of the Second Session of the 13th National Committee of the CPPCC (No. 085 of Medical and Sports Category)*. http://www.nhc.gov.cn/wjw/tia/202009/84ffa320a2e943b0b0b8c80baebf2621.shtml (2020).

[19] Publicity Department of the National Health Commission. *Transcript of the Press Conference of the National Health Commission on May 30, 2022*. http://www.nhc.gov.cn/xcs/s3574/202205/71ecabbcfa8f46ec920f1b7545cf02f0.shtml (2022).

[20] Chen CY, Huang JY, Chen XY, Wang M (2021). Epidemiological characteristics of hepatitis C in Zhongshan City, 2005–2019. Chin. J. Health Educ..

[21] Ma J (2021). Meta-analysis on the positive rate of hepatitis C antibody among pregnant females in China from 2008 to 2018. Chin. J. Evid. Based Med..

[22] Ding GW (2019). Sentinel surveillance for viral hepatitis C in China, 2016–2017. Chin. J. Epidemiol..

[23] Bigna JJ (2019). Gender development and hepatitis B and C infections among pregnant women in Africa: a systematic review and meta-analysis. Infect. Dis. Poverty.

[24] Ahmad I (2016). Prevalence of hepatitis B and C viral infection among pregnant women in Peshawar, Pakistan. Hepat. Mon..

[25] Khamis HH, Farghaly AG, Shatat HZ, El-Ghitany EM (2016). Prevalence of hepatitis C virus infection among pregnant women in a rural district in Egypt. Trop. Doct..

[26] Costa ZB (2009). Prevalence and risk factors for Hepatitis C and HIV-1 infections among pregnant women in Central Brazil. BMC Infect. Dis..

[27] Dagnew M (2020). Hepatitis B and C viruses' infection and associated factors among pregnant women attending antenatal care in hospitals in the Amhara National Regional State Ethiopia. Int. J. Microbiol..

[28] Sarah KD-K, Jeffrey AK, Brenna LH (2021). Society for maternal-fetal medicine consult series #56: Hepatitis C in pregnancy—updated guidelines: Replaces Consult Number 43, November 2017. Am. J. Obstet. Gynecol..

[29] Smith DD, Rood KM (2020). Intrahepatic Cholestasis of pregnancy. Clin. Obstet. Gynecol..

[30] Marschall HU, Wikström Shemer E, Ludvigsson JF, Stephansson O (2013). Intrahepatic cholestasis of pregnancy and associated hepatobiliary disease: A population-based cohort study. Hepatology.

[31] Rouabhia S (2010). Prevalence of type 2 diabetes in Algerian patients with hepatitis C virus infection. World J. Gastroenterol..

[32] Piffer S, Mazza A, Dell'Anna L (2020). Serological screening for hepatitis C during pregnancy: Seroprevalence and maternal and neonatal outcomes in 45,000 pregnant women. Eur. J. Obstet. Gynecol. Reprod. Biol..

[33] Connell LE (2011). Maternal hepatitis B and hepatitis C carrier status and perinatal outcomes. Liver Int..

[34] Parent S, Salters K, Awendila L, Ti L (2018). Hepatitis C and pregnancy outcomes: A systematic review protocol. BMJ Open.

[35] Ragusa R (2020). Hepatitis C virus infection in children and pregnant women: An updated review of the literature on screening and treatments. AJP Rep..

[36] Benhammou V (2018). HBV or HCV coinfection in HIV-1-infected pregnant women in France: prevalence and pregnancy outcomes. J. Acquir. Immune Defic. Syndr..

[37] Money D (2014). Obstetrical and neonatal outcomes among women infected with hepatitis C and their infants. J. Obstet. Gynaecol. Can..

[38] Zhan ZW, He RH, Lin HY, Yuan WS, Li Q (2019). Hepatitis C virus positive unpaid blood donors with hepatitis B virus and human immunodeficiency virus treponema pallidum infection in Zhongshan City, Guangdong Province. J. Pract. Med. Tech..

[39] Mutagoma M (2017). Hepatitis C virus and HIV co-infection among pregnant women in Rwanda. BMC Infect. Dis..

